# Distinct transcriptomic strategies underlie differential heat tolerance in Symbiodiniaceae symbionts

**DOI:** 10.1093/ismejo/wraf268

**Published:** 2025-12-04

**Authors:** Tingting Xiang, Stephanie L Peak, Eric C Huitt, Arthur R Grossman

**Affiliations:** Department of Bioengineering, University of California, Riverside, Riverside, CA 92521, United States; Department of Bioengineering, University of California, Riverside, Riverside, CA 92521, United States; Department of Biology, University of North Carolina at Chapel Hill, Chapel Hill, NC 27599, United States; Department of Bioengineering, University of California, Riverside, Riverside, CA 92521, United States; Division of Biosphere Sciences and Engineering, Carnegie Institution for Science, Stanford, CA 94305, United States; Department of Biology, Stanford University, Stanford, CA 94305, United States

**Keywords:** Symbiodiniaceae, thermal stress, genome stability, DNA repair, proteasome, photosynthesis, transcriptomics, symbiosis

## Abstract

Dinoflagellate algae in the family Symbiodiniaceae, symbionts of many marine cnidarians are critical for the metabolic integrity of reef ecosystems, which are increasingly threatened by environmental stress. The resilience of the cnidarian-dinoflagellate symbiosis depends on thermotolerance of the partner organisms; coral hosts that harbor heat-resistant symbionts exhibit greater resistance to bleaching. Although coral responses to heat stress are well-documented, transcriptomic adaptation/acclimation of Symbiodiniaceae to elevated temperatures are limited. Here, we compare thermal responses of two species representing two genera of Symbiodiniaceae, *Symbiodinium linucheae* (strain SSA01; ITS2 type A4) and *Breviolum minutum* (strain SSB01; ITS2 type B1). SSA01 in culture maintained photosynthetic function at elevated temperatures and mounted a rapid transcriptomic response characterized by early downregulation of a JMJ21-like histone demethylase coupled with prompt upregulation of transcripts associated with DNA repair and oxidative stress, which would likely contribute to enhanced resilience to heat stress. In contrast, SSB01 experienced a decline in photosynthetic efficiency and a delayed transcriptomic response that included upregulation of transcripts encoding proteasome subunits and reduced transcripts encoding proteins involved in photosynthesis and metabolite transport. These findings indicate that a rapid and moderate transcriptomic response that results in increased expression of genes related to the synthesis and repair of biomolecules might be crucial for thermal tolerance in the Symbiodiniaceae whereas sensitivity to elevated temperatures may be reflected by increased protein turnover and a marked decline in anabolic processes. Understanding these differences is vital for predicting coral responses to warming seas and developing strategies to mitigate heat-stress impacts on reefs.

## Introduction

Dinoflagellates are single-celled eukaryotic algae that are abundant in nature, serve a variety of ecosystem functions, and lead lifestyles ranging from that of a planktonic photosynthetic autotroph to a heterotrophic parasite [1, 2]. Dinoflagellates in the family Symbiodiniaceae form symbiotic relationships with cnidarians including corals, jellyfish, and sea anemones. These algae provide the metabolic foundation of coral reef ecosystems [3–5]. They populate host gastrodermal tissue (inner cell layer of the gut and tentacles) as endosymbionts, harnessing the power of sunlight to drive photosynthesis, with most of the photosynthate translocated to the host [3, 5, 6]. In return, the symbionts receive inorganic nutrients (e.g. nitrogen, sulfur, carbon, and phosphorus) from the host as well as protection from predation [5, 7]. This metabolic mutualism is the foundation of healthy coral ecosystems, as the algae provide their coral/cnidarian hosts with a majority of the energy that they need to thrive [3, 4]. With global warming and increasing sea water temperatures, the coral-Symbiodiniaceae association is compromised and algal symbionts vacate the host tissue; this stress response is known as coral bleaching [8, 9]. Prolonged bleaching events can result in mass coral mortality that devastates reef ecosystems [5, 10]. Despite the importance of cnidarian-Symbiodiniaceae symbiosis for the preservation of reef ecosystems, little is understood of the molecular mechanisms that govern the establishment and degradation of this relationship.

The Symbiodiniaceae represent a highly diverse family of algae composed of species that have evolved varying levels of heat tolerance [11, 12]. For the coral-Symbiodiniaceae association, the thermal tolerance of the alga contributes to reef resilience when confronted with elevated ocean temperatures [13–16]. Several Symbiodiniaceae species, including *Durusdinium trenchii* and *Cladocopium thermophilum*, are relatively heat-tolerant and have been associated with survival of coral holobionts at elevated temperatures [17–19]. Additionally, in both field and laboratory experiments, corals dominated by *D. trenchii* symbionts were often found to be more resistant to bleaching than corals dominated by symbionts of the genera *Breviolum* or *Cladocopium* [20–22]. Heat-sensitive strains are more susceptible to a loss of photosynthetic activity as the temperature increases than heat tolerant strains, which may reflect the stability of photosynthetic complexes and antennae protein interactions, thylakoid membrane composition and structure [23–25] and various other cellular processes. Although extensive physiological studies have characterized a variety of responses associated with Symbiodiniaceae thermal stress [12, 26–30], investigations of these responses at the level of transcription are limited. As a result, molecular mechanisms underlying differential thermal tolerances of various Symbiodiniaceae species are still largely underexplored.

In this study, we analyzed the physiological and transcriptomic responses of two Symbiodiniaceae species, *Symbiodinium linucheae* (clonal, axenic strain SSA01 [31]) and *Breviolum minutum* (clonal, axenic strain SSB01 [32]), to short- and long-term heat stress. These strains were originally isolated from the symbiotic sea anemone *Exaiptasia diaphana* (here referred to as Aiptasia) and were observed to have divergent thermal responses, with strain SSA01 being more thermally tolerant than SSB01 [12]. Here we show that Aiptasia populated with the thermally sensitive SSB01 exhibited more rapid and severe bleaching when exposed to heat stress (34°C) than Aiptasia hosting the thermally resistant endosymbiont SSA01. In culture, heat-stressed (34°C) SSB01 displayed a marked reduction in chlorophyll fluorescence, whereas heat-exposed SSA01 exhibited only a small change in chlorophyll fluorescence. Additionally, photosynthetic efficiency was drastically reduced in SSB01 after 4 days of heat stress at 34°C, whereas it was relatively unaffected in SSA01 over the 8-days of the experiment. At the genomic level, SSA01 exhibited a rapid heat triggered transcriptional response with activation of genes encoding proteins involved in genome stability, repair processes and the synthesis of biomolecules. In contrast, thermally sensitive SSB01 showed small transcriptional changes during short-term heat exposure, but after 3 days there were pronounced increases in the levels of transcripts related to proteolysis and catabolism of biomolecules, and reduced levels of transcripts related to ion transport, metabolism of carbohydrates, and photosynthesis. Overall, our data suggest that a slow transcriptional response to heat stress followed by prolonged, extensive differential expression of genes associated with cellular stress and protein turnover may be linked to heat sensitivity in the Symbiodiniaceae, whereas the ability to mount a rapid transcriptomic response focused on maintenance of genome stability, repair, and the amelioration of the stress conditions may enable the cells to cope with the new conditions and minimize degradation of vital cellular processes.

## Materials and methods

### Organisms and culture conditions

Symbiodiniaceae cultures were maintained as described [33]. Clonal and axenic *Breviolum minutum*, strain SSB01 (Clade B), and *Symbiodinium linucheae*, strain SSA01 (Clade A), were used in this study. SSB01 and SSA01 liquid cultures were grown in 37.4 g·L^−1^ marine broth (MB) (Millipore-Sigma 76 448) medium without agitation. Prior to thermal stress exposure, cultures were maintained at 27°C on a 12 h-light/12 h-dark cycle with an irradiance of ~10 μmol photons m^−2^ s^−1^ of photosynthetically active radiation (PAR) provided by Percival SciWhite LED tiles.

Clonal *Exaiptasia diaphana* (referred to as Aiptasia) strain CC7 sea anemones used in this study were rendered aposymbiotic using a short-term cold shock method [34] and menthol bleaching [35]. Aposymbiotic anemones were confirmed to be free of symbiotic algae by fluorescence microscopy after long term growth in the absence of symbionts. Anemones were maintained in artificial seawater (ASW) in polycarbonate containers at 27°C on a 12 h-light/12 h-dark cycle (25 μmol photons m^−2^ s^−1^ PAR from Percival SciWhite LED panels). Aiptasia were fed Artemia, with the medium in the growth tanks replaced twice per week. Inoculated anemones were allowed to reach a stable algal density over 30 days and were starved 2 days prior to initiating temperature stress experiments.

### Temperature treatment experiments

To examine the effects of elevated temperature on cultured Symbiodiniaceae species, ~5x10^6^ SSB01 and SSA01 cells from log phase cultures (density 10^6^ cells/ml) were exposed to either ambient (27°C) or elevated (34°C) temperatures over a period of 8 d under a 12 h-light/12 h-dark cycle (~10 μmol photons m^−2^ s^−1^ PAR from Percival SciWhite LED panels).

For *in hospite* temperature stress studies, Aiptasia CC7 populated with SSB01 or SSA01 (SSB01-CC7 and SSA01-CC7, respectively) were exposed to either continuous ambient (27°C) or elevated (34°C) temperatures without being fed for 8 d. Inoculated anemones were kept individually in 6-well polypropylene plates under a 12 h-light/12 h-dark regime (25 μmol photons m^−2^ s^−1^ PAR provided by Percival SciWhite LED panels) and were monitored during the temperature treatments using a Nikon SMZ-25 fluorescence stereoscope either in bright-field or fluorescence modes. To monitor chlorophyll fluorescence from the endosymbiont, the anemones were exposed to blue-light excitation (absorbed by chlorophyll) and fluorescence emission monitored in the red region of the spectrum (GFP2 filter set; 480/40 nm excitation, 510 nm long-pass emission); fluorescence images were captured using a Nikon DS-Ri2 Color CMOS Camera.

### Algal fluorescence profiles using guava flow cytometry

To determine the health of the cultured algal cells during temperature treatments, SSB01 and SSA01 were collected, and after exposure to 34°C for 0, 1, 2, 3, 4, and 8 d, were analyzed using Guava flow cytometry (Guava easyCyte HT 2 laser flow cytometer: EMD Millipore) as previously described [32, 36]. The Guava limits (window) for defining healthy cells were set using a combination of side scatter and chlorophyll fluorescence prior to being exposed to the elevated temperature (day 0). Reduction in chlorophyll fluorescence and pigment loss occurred as the cells experienced stress resulting from the elevated temperature conditions.

### Analyses of photosynthetic function

Chlorophyll *a* content was determined spectrophotometrically following pigment extraction in 100% methanol [37]. The maximum quantum efficiency of PSII (*F*_v_/*F*_m_ = (*F*_m_-*F*_0_/*F*_m_) was used to evaluate photosynthetic function of the cultured algae and was measured with a Dual PAM-100 fluorometer (Heinz Walz) as previously described [38]. Cell samples (~10^6^ cells) were maintained in the dark for 30 min with continuous agitation prior to making the measurements.

### Symbiont density quantification in Aiptasia

Symbiont density in Aiptasia was quantified by normalizing algal cell counts to host protein content. Individual anemones were homogenized in ultrapure water containing 0.01% SDS using a Tissue Ruptor (Fisher PowerGen125) for 8–10 s until no visible tissue fragments remained. Homogenates were mixed thoroughly, and aliquots were taken for symbiont enumeration using a Countess 3 FL Automated Cell Counter and for host protein quantification using the Pierce BCA Protein Assay Kit, following the manufacturers’ instructions. Symbiont density was calculated as algal cells per μg host protein to account for variation in host biomass.

### RNA-seq analyses

#### RNA extraction and sequencing

SSA01 and SSB01 cells grown to exponential phase at 27°C were exposed to 34°C and samples were collected at 0 h, 3 h, 12 h, 1 d, 2 d, 4 d, and 8 d. Samples were also collected from cultures maintained at 27°C for 8 days to serve as a temperature control. Total RNA was extracted from the cells using the phenol/chloroform method [38, 39]. Each RNA sample was assessed using an Agilent 2100 Bioanalyzer, and only samples with RNA-integrity scores ≥8 were used for library preparations. Approximately 1 μg of total RNA per sample was processed according to the TruSeq RNA Sample Prep Kit (Illumina FC-122–1001) to construct the cDNA libraries. The resulting libraries were pooled based on their indices according to kit instructions, with sequencing and clustering of the 101-bp paired-end reads performed on a HiSeq 2000 System (Illumina) by the Stanford Center for Genomics and Personalized Medicine. All raw sequencing reads are available in the Sequence Read Archive PRJNA591730.

#### De novo *transcriptome assembly and annotation for SSA01*

To ensure that high-quality data were used for the transcriptome assembly, SSA01 reads were filtered for quality and length. Low-quality bases were trimmed using Trimmomatic [40] such that no nucleotide had a quality score of less than 10 and no ambiguous nucleotides (N’s) remained. Any remaining polyA sequences were removed using Cutadapt [41]. After trimming the reads, all reads of 45 bases or fewer were eliminated. The remaining reads were used for *de novo* assembly of the SSA01 transcriptome using Trinity (version 2.3.2) [42]. Minimum transcript length for *de novo* assembly was set to 200 bp. To identify the longest isoforms among the various alternative transcripts, the filter_longest_trinity_subcomponents.py script from the Trinity toolkit was employed. Assembly completeness was assessed using BUSCO v3.0.2 in transcriptome mode with the eukaryota_odb9 dataset. The SSA01 transcriptome recovered 80.5% complete BUSCOs (71.3% single-copy, 9.2% duplicated), 4.3% fragmented, and 15.2% missing of the 303 expected orthologs, indicating a high level of completeness. The assembled transcriptome has been deposited at DDBJ/ENA/GenBank under the accession number GLGU00000000; the version reported here is GLGU01000000.

The Blast2GO suite version 4.0.7 (http://www.blast2go.com) was used to analyze the SSA01 transcriptome. Putative protein products were assigned with transcripts based on BLASTX, with an E-value cut-off of 0.00001. Gene Ontology terms were assigned based on BLASTX hits in the species-specific entries of the gene-product table of the GO database. We used the 'generic' GOSlim mapping term (goslim_generic.obo) available in BLAST2GO and InterPro annotation to identify conserved protein domains.

#### Gene expression analysis

The *de novo* SSA01 transcriptome assembly contained 71 057 contigs (79.5 Mb total, mean length 1118 bp; N50 = 1697 bp). Over 90% of quality-filtered reads mapped back to the SSA01 assembly, indicating high completeness and minimal assembly artifacts. As Symbiodiniaceae genomes are incomplete and highly repetitive [43], reads were mapped to this transcriptome for differential expression analyses. RNA-seq reads from a total of 27 SSA01 and 26 SSB01 libraries (Supplementary Table S1) were aligned to the reference SSA01 and SSB01 (Symb6 [38]) transcriptomes, respectively, using the BWA software package [44]. The number of reads that aligned to each transcript with a mapping quality score of >30 was counted using samtools. Statistical differences in the level of gene expression were evaluated using the R package DESeq2 [45, 46], with transcripts considered differentially expressed having a Benjamini–Hochberg false discovery rate (FDR)-adjusted *P* value ≤0.001. Sixteen pairwise comparisons were made, with each time point for SSA01 and SSB01 compared to their 0 h time point, respectively. Comparisons were performed to determine gene expression patterns at the different times of exposure to 34°C, as well as at the 8 d control condition maintained at 27°C. The integrity of transcripts used in differential expression analyses was verified based on read coverage and open reading frame consistency, with no evidence of chimeric or artifactual assembly. These checks confirm that the transcriptome-based approach reliably represents true gene expression patterns.

#### K-means clustering

K-means clustering was performed using KMeans implementation from the scikit-learn library in Python to categorize gene expression profiles. Significantly altered transcripts from SSA01 and SSB01 were clustered separately, resulting in eight distinct expression clusters for each strain.

#### Ortholog identification in SSA01 and SSB01

To identify SSA01 and SSB01 orthologs, we employed two complementary methodologies. First, we searched for orthologs using ggsearch, a component of the Fasta3 package that implements an algorithm derived from the Needleman and Wunsch algorithm [47]. Additionally, reciprocal BLASTP analyses were conducted, applying an E-value cutoff of 1 × 10^−10^ for both directions. The results from both approaches were subsequently merged to establish a set of orthologous sequences in SSA01 and SSB01 (Dataset S4).

## Results and discussion

### *Symbiodinium linucheae* SSA01 is heat tolerant whereas *Breviolum minutum* SSB01 is heat sensitive both *in culture* and *in hospite*

**Figure 1:**
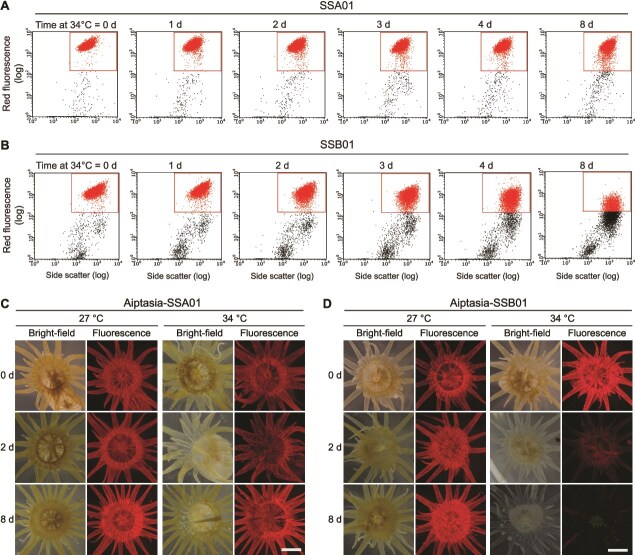
*Symbiodinium linucheae* SSA01 is heat tolerant whereas *Brevolium minutum* SSB01 is heat sensitive in culture and *in hospite*. (A and B) Guava flow cytometry profiles of cultured *S. linucheae* SSA01 (A) and *B. Minutum* SSB01 (B) cells based on chlorophyll fluorescence and side-scatter following maintenance of the cultures at 34°C over an 8-d period. (C and D) Representative images (from above) of *Exaiptasia pallida* (Aiptasia) populated with (C) SSA01 and (D) SSB01 algal symbionts. Animals were maintained in 12 h light/12 h dark at 27°C (left panels) or 34°C (right panels). Bright-field and algal chlorophyll autofluorescence (red) is shown from d-0, d-2, and d-8 after raising the temperature.

To investigate the responses of Symbiodiniaceae to heat stress, clonal, axenic *Symbiodinium linucheae* (strain SSA01) and *Breviolum minutum* (strain SSB01) were exposed to an elevated temperature of 34°C for 8 days. We monitored the fluorescence profiles of these and control cultures (maintained at 27°C) using the Guava flow cytometer, which reports levels of chlorophyll fluorescence. The fluorescence intensity window was set to capture nearly all 27°C-grown unstressed SSA01 and SSB01 cells (Fig. 1A and B, S1; prior to raising the temperature or time 0). For SSB01 the chlorophyll fluorescence levels gradually declined over the 8 d of exposure to elevated temperature (Fig. 1B); at 8 d, SSB01 showed a marked decline in chlorophyll fluorescence intensity under heat stress, with approximately half of the population falling outside the normal detection window compared to cells maintained at 27°C. In contrast, the chlorophyll fluorescence of SSA01 cells exhibited little or no decline over the entire period of exposure to 34°C (Fig. 1A).

Both SSB01 and SSA01 form symbiotic relationships with various cnidarian hosts [31, 32]. Hence, we determined whether the responses of cultured Symbiodiniaceae to heat stress reflected the sensitivity of the cnidarian-Symbiodiniaceae association. Aiptasia that was fully populated with SSA01 (Aiptasia-SSA01) or SSB01 (Aiptasia-SSB01) were maintained at 27°C or exposed to 34°C for 8 d (Fig. 1C and D). We tracked the symbionts in individual sea anemones maintained at 27°C or 34°C and observed a rapid decline in fluorescence in anemones symbiotic with SSB01 (Aiptasia-SSB01) over the 8 d of heat exposure (Fig. 1D). Quantitative symbiont density data (Fig. S1) corroborate that this decline was primarily due to the loss of the algae from the host tissue. In contrast, SSA01 was maintained at near constant levels in the anemones over the entire heat treatment (Fig. 1C). When maintained at 27°C, the anemones associated with either of the algal species exhibited no or little decline in their algal population over the same period (Fig. 1C and D).

### S‌SA01 displays rapid transcriptional responses to elevated temperature whereas SSB01 responses are gradual

**Figure 2:**
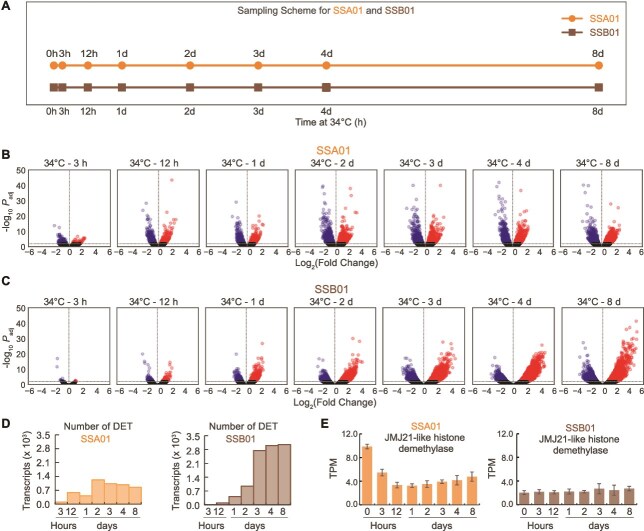
*Symbiodinium linucheae* SSA01 launches a rapid transcriptional response whereas *Breviolum minutum* SSB01 gradually alters its transcriptome after elevating the temperature. (A) Sampling scheme for RNA-Seq analysis showing the time points at which samples from the SSA01 and SSB01 cultures were collected following their transfer to 34°C. (B and C) Volcano plots of relative abundances of individual transcripts in (B) SSA01 and (C) SSB01. °C-axis, fold-changes; *y*-axis, adjusted *P* values based on Benjamini-Hochberg correction. Both axes use log scales. The horizontal line toward the bottom of each plot represents an adjusted *P* value threshold of 0.001, which is the cutoff used to determine statistical significance. (D) The number of transcripts that significantly changed (DET; differentially expressed transcripts) in the transcriptomes of SSA01 and SSB01 at each time point following the transfer of cultures to 34°C relative to time 0 h (just prior to raising the temperature). (E) Transcript abundance (TPM) of a JMJ21-like histone demethylase in SSA01 (left) and SSB01 (right) across the short- and long-term heat stress time course (0 h to 8 d).

Changes in the transcriptome patterns of SSA01 and SSB01 following exposure of cultured cells to 34°C were probed using RNA-seq. Transcriptome profiles of both strains were captured over the short- and long-terms (≤24 and > 24 h), with samples taken at 3 h, 12 h, 1 d, 2 d, 3 d, 4 d, and 8 d (Fig. 2A) following exposure of cells to 34°C. Differentially expressed transcripts (DETs) were identified by comparing transcript levels at each time point following heat exposure to those in 27°C-grown cells (time 0; prior to high temperature exposure). A stringent adjusted *P* value threshold (based on Benjamini-Hochberg correction) of ≤0.001 was applied to identify high-confidence DETs and ensure rigorous control of false positives (Dataset S1, S2). A similar stringency was adopted in recent Symbiodiniaceae and coral transcriptomic studies [7, 48–50]. To control for time-dependent effects unrelated to heat stress, RNA was analyzed from parallel cultures of SSA01 and SSB01 maintained at 27°C for 8 d; at the 8-d time point, transcriptome changes for both algae were shown to be minimal, with small fluctuations limited to a few transcripts (Fig. S2A and B). After a 12 h exposure of SSA01 to the elevated temperature, 584 DETs were identified (Fig. 2B, D), and after 2 d the number of DETs was approximately double that of the 12-h time point. Some decrease in the total number of DETs occurred between 2 d and 8 d (Fig. 2B, D). In contrast, SSB01 cells experienced very limited changes in their transcriptome over the first 12 h of exposure to 34°C. However, the number of SSB01 DETs gradually increased following exposure of the cells to the elevated temperature, surpassing the number of DETs determined for SSA01; 1033 DETs for SSA01 and 2753 DETs for SSB01 after 3 d at 34°C. The number of SSB01 DETs continued to rise, with detection of 3054 DETs after 8 d at 34°C, which is more than 3-fold higher than that of SSA01 (Fig. 2C, D). These results indicate that SSA01 algae mount a rapid transcriptional response to heat stress compared to SSB01, which may be linked to the thermal tolerance of SSA01. The elevated levels of DETs in SSB01 over the long term may reflect stress responses and dramatic changes in the cell’s physiology.

To assess whether SSA01 displays a transcriptionally pre-adapted state, we analyzed SSA01 transcript levels, measured as transcripts per million (TPM), for DETs across short-term time points (3 h, 12 h, and 1 d). We did not consistently observe elevated transcript abundances in SSA01 relative to SSB01 during this period (Fig. S2C). To further evaluate whether SSA01 expresses genes prior to heat exposure that may contribute to thermal resilience, we compared TPM values at 0 h between the two species for orthologous transcripts corresponding to SSA01 DETs (*P* <  0.001) (Fig. S2D) and for SSA01 orthologs of SSB01 heat-elevated genes identified after short-term heat stress (3 h, 12 h, and 1 d) (Fig. S2E). We identified 164 transcripts with higher expression in SSA01 relative to SSB01 (Fig. S2D). This pattern of elevated baseline expression, often referred to as transcriptional front-loading, included genes enriched for oxidoreductase activity, DNA/RNA binding, and homeostatic processes. The transcripts included those encoding peroxiredoxin, the UVB photoreceptor UVR8, and cold shock-like proteins (Dataset S3); these stress-responsive factors could contribute to a preconditioned state of SSA01 that would afford the cells greater heat tolerance relative than SSB01. Additionally, 38 of the most elevated genes in SSB01 during heat stress (Log₂FC > 2) were already more abundant in SSA01 prior to heat exposure (0 h) (Fig. S2F). These also represent front-loaded genes, and include *Regulator of nonsense transcripts 1-like*, which plays a role in mRNA quality control [51], potentially providing SSA01 with a thermal advantage through the removal of faulty transcripts.

We observed decreased abundance of a transcript encoding the JMJ21-like histone demethylase (SSA01_DN16561_c0_g1_i3; SSB01 ortholog: s6_42821) in SSA01 within 12 h of exposure of the cultures to the elevated temperature (Fig. 2E). This gene encodes a member of the Jumonji C (JmjC) domain-containing histone demethylase family, known to remove repressive H3K27me3 marks and regulate gene activation, chromatin remodeling, and genome stability [52–54]. In contrast, SSB01 maintained consistently low levels of the JMJ21-like histone demethylase transcript throughout the time course; the levels were comparable to, or even lower than those observed in SSA01 after 12 h of heat exposure. As SSA01 mounted an early transcriptional adjustment (first 12 h of heat exposure), the rapid decline in expression of the JMJ21-like demethylase may reflect a strategic transition toward transcriptional restraint, limiting further chromatin remodeling and widespread gene activation. In addition to controlling transcriptional responses, JmjC domain demethylases contribute to genome stability by maintaining histone and DNA methylation homeostasis and preventing transposon activation [54–56]. Furthermore, recent studies have shown that in dinoflagellates, large-scale genome organization is highly sensitive to transcriptional activity and is shaped by transcription-induced supercoiling rather than conventional chromatin looping [57, 58]. Thus, downregulation of the JMJ21-like histone demethylase-like gene in SSA01 may serve a dual function: stabilizing gene expression to avoid excessive transcriptional remodeling, and protecting the three-dimensional genome architecture under thermal stress. In contrast, multiple *Regulator of Chromosome Condensation 1* (*RCC1*)-family genes were upregulated in SSB01, whereas no such response was detected in SSA01 (Fig. S2G, Dataset S1, S2), suggesting that SSB01 may engage a distinct and potentially compensatory mechanism to modulate chromosome organization under heat stress. Consistent with this theme, *Fugacium kawagutii*, which exhibits intermediate sensitivity to heat stress [59], also upregulates *RCC1* genes under heat stress [60]. In other eukaryotic systems, aberrant regulation of RCC1 has been associated with genomic instability and altered cell-cycle progression through its effects on key checkpoints, transcription factors, and cell-cycle regulators [61]. Together, these contrasting chromatin-associated responses potentially contribute to the enhanced transcriptomic stability in SSA01 and the broader transcriptional disruption observed in SSB01 under heat stress (discussed below). These findings suggest that transcriptional front-loading, followed by early transcriptional adjustments and chromatin and genome stabilization may be key features of thermal tolerance in SSA01.

Dinoflagellates possess highly derived, noncanonical histones and atypical chromatin organization [38, 57, 58, 62–65], raising uncertainty about whether histone-modifying enzymes function as they do in model eukaryotes. The functional annotation of the *JMJ21*-like gene, and others discussed throughout this study, is based on conserved domain homology, and thus functional inference should be viewed with caution. Because targeted genetic manipulation in Symbiodiniaceae remains technically challenging [33, 66], these annotations are best regarded as hypotheses about potential roles rather than definitive functional assignments.

### Temporal dynamics of gene expression in response to heat stress

**Figure 3:**
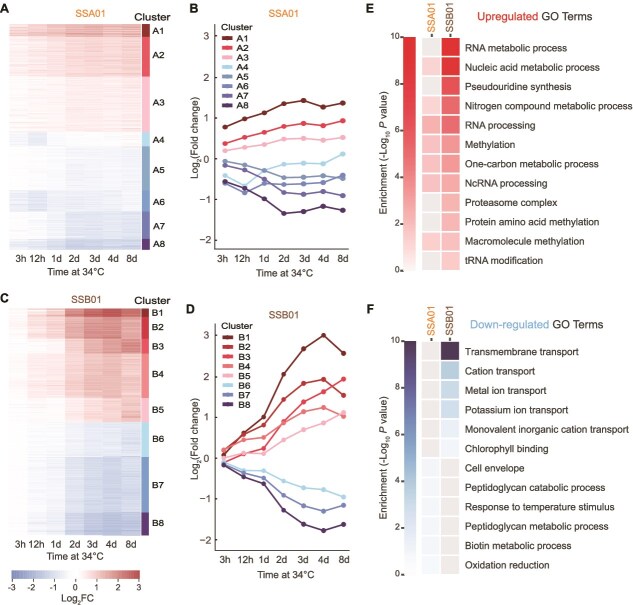
Transcriptomic responses and functional enrichment in SSA01 and SSB01 cells exposed to elevated temperatures for 8 days. Heatmap depicting transcripts that differentially accumulate in SSA01 (A) and SSB01 (C) during an 8-d exposure to 34°C (from 27°C growth). In total, 4196 DETs were identified in SSA01 and 11 108 DETs in SSB01. Every horizontal row represents an individual transcript. K-mean clustering of the transcripts resulted in 8 different expression categories over the 8 d of exposure to 34°C (B, D). *x*-axis, time at 34°C; *y*-axis, fold-change. Heatmap showing *P* value for the enrichment of each GO category (based on hypergeometric *P* value < 0.1) for the up-regulated (E) and down-regulated (F) transcripts that exhibit significant differences in their abundances. Gray colored boxes indicate not significant.

K-means clustering was used to delineate temporal patterns of gene expression over time for both SSA01 and SSB01 (Figs 3A–D). For SSA01, some clusters exhibited differential expression within the first 12 h of heat exposure, with subsequent return to baseline levels over the experimental period. The transcripts of other SSA01 clusters exhibited a gradual change in abundance, stabilizing at an ~2-fold difference relative to the level of cells at time zero; this includes genes that are up- or down-regulated (Fig. 3A and B). In contrast, changes in accumulation of SSB01 transcripts were delayed following the transfer of cells to elevated temperature, with expression patterns not markedly diverged until the cells were exposed to 34°C for >1 d; the number of differentially expressed genes increased over the entire 8 d of heat exposure. Unlike SSA01, expression levels of the genes in these clusters did not return to baseline and often attained ~4-fold levels of upregulation and downregulation (Fig. 3C and D).

### S‌SB01 displays a “stressed” transcriptome under elevated temperature

We applied Gene Ontology (GO) enrichment analysis to explore the functional categories of both the up-regulated (Fig. 3E) and down-regulated (Fig. 3F) DETs in SSB01 and SSA01 during short-term and long-term exposure to 34°C. In SSB01, GO terms associated with “RNA metabolic process”, “Nucleic acid metabolic process”, ``Proteasome complex'' and ``Pseudouridine synthesis'' were enriched among the upregulated genes (Fig. 3E), whereas terms related to “Transmembrane transport,” including cation transport, and “Chlorophyll binding” were enriched among the downregulated genes (Fig. 3F).

### Proteasomal subunit dynamics in SSB01 under thermal stress

**Figure 4:**
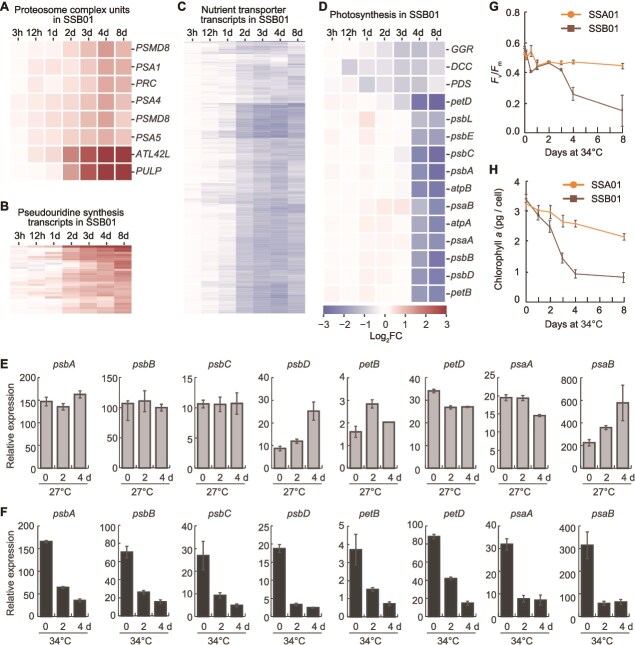
Differential expression of proteasome complex and photosynthesis genes in SSB01 under thermal stress. (A–D) Heat maps showing the expression levels (fold change at 34°C relative to 27°C) of transcripts encoding proteasome complex subunits (A), pseudouridine synthesis proteins (B), substrate-specific transporters (C), and proteins involved in photosynthesis (D). Each row represents an individual transcript. (E and F) Relative gene expression levels of representative chloroplast genes measured by qPCR in SSB01 at various times at 27°C (E) and following transfer to 34°C (F). The values are normalized to a reference gene and shown as mean ± SD across three biological replicates. (G) Maximum PSII efficiency (*F*_v_/*F*_m_) of SSB01 and SSA01 grown in 12 h light/12 h dark at 34°C. Error bars represent ±SD from at least three biological replicates. (H) Chl *a* concentrations in SSB01 and SSA01 grown in 12 h light/12 h dark at 34°C. Error bars represent ±SD from at least three biological replicates.

Six transcripts encoding proteins integral to the 26S proteasome complex, the predominant proteolytic system in eukaryotic cells, exhibited an increase in abundance in SSB01 of more than 1.5-fold beginning on day 4 following exposure to 34°C. These transcripts include s6_38597 (26S proteasome non-ATPase regulatory subunit 8, *PSMD8*); s6_38340 (proteasome subunit alpha 1, *PSA1*); s6_42720 (proteasome regulatory component, *PRC*); s6_13121 (proteasome subunit alpha type subunit 4, *PSA4*); s6_56095 (26S proteasome non-ATPase regulatory subunit 8, *PSMD8*); and s6_4825 (proteasome subunit alpha 5, *PSA5*). Transcripts associated with the ubiquitin system were also elevated. Specifically, transcripts encoding E3 ubiquitin-protein ligase atl42-like (s6_55036, *ATL42L*) and a putative ubiquitin ligase protein (s6_42574, *PULP*), both associated with protein ubiquitination, were more than 20-fold elevated on d-8 relative to 27°C control cells (Fig. 4A). In contrast, transcripts in the same proteasome-related functional category showed no significant enrichment or differential expression in SSA01 (Fig. 3E).

### Pseudouridine synthesis

Transcripts encoding enzymes associated with pseudouridine synthesis were also upregulated (≥2-fold in abundance) in SSB01 during long-term heat stress, with one such transcript showing a ≥8-fold change (s6_47859) after 4 d at 34°C (Fig. 4B). Additionally, transcript s6_43192, encoding a pseudouridylate synthase 7 (PUS7) homolog, exhibited a ~4-fold increase following 8 d of elevated temperature. PUS7 catalyzes isomerization of specific uridine residues to pseudouridine in RNAs. This modification can increase the rigidity of the RNA backbone (through introduction of a C-C bond) and frees the N1 position for additional hydrogen bonding. These alterations can lead to enhanced stability of pseudouridine-adenine compared to uridine-adenine base pairs [67], increased stability and translational efficiency of the mRNAs, and altered RNA–RNA and RNA–protein interactions, especially under stress conditions [68, 69], including heat stress [70]. The coordinated upregulation of pseudouridine synthase genes—including those encoding ribosomal large subunit pseudouridine synthase D and tRNA pseudouridine synthase A—coincides with widespread transcriptomic disruption in SSB01, suggesting a role for pseudouridylation in preserving RNA stability, function, and/or translational capacity during prolonged heat stress. It is known that transcripts from algal plastids exhibit 3′ polyuridylylation, and genes involved in pseudouridine synthesis were upregulated in SSB01 in response to glucose-induced stress, which led to chloroplast degradation and strong downregulation of chloroplast gene expression [37]. However, these pseudouridylation genes are not differentially expressed in this heat stress dataset. This indicates that pseudouridylation may be a component of the stress response in the Symbiodiniaceae that functions under distinct physiological perturbations, including prolonged thermal or metabolic stress.

### Downregulation of transport-related transcripts in SSB01 during thermal stress

Exposure to 34°C led to a marked downregulation of transcripts associated with metabolite/cation transport in SSB01 (Fig. 4C). Following 2 d of heat stress, there was a significant reduction in the levels of transcripts encoding transmembrane transporters, including calcium ion-binding proteins and metal-ion transporters. For example, the levels of transcripts encoding a urea transporter (s6_2203) and putative ATP-binding cassette (ABC) transporters (s6_4229 and s6_29617) decreased by more than fourfold after 4 d of heat stress. The pronounced downregulation of the urea transporter was part of a broader suppression of nitrogen transport capacity, with nearly all ammonium and nitrate transporters showing reduced expression during prolonged heat stress (Fig. S3). This widespread repression suggests a concerted reduction in the potential for nitrogen assimilation at the elevated temperature, consistent with evidence that heat stress destabilizes coral–algal nutrient cycling by shifting the holobiont from nitrogen to carbon limitation [71]. Such transcriptional responses may represent a conserved strategy to minimize energy-demanding nutrient uptake as cells experience thermal and redox stress. Expression of numerous potassium transporters that participate in both active and passive potassium transport (s6_10943, s6_12784, s6_2370, s6_8003, s6_29869, s6_6724, s6_28285, s6_13287, s6_13461, s6_19586) declined to nearly 50% of the level observed in cells maintained at 27°C. Similar expression patterns were observed for sodium (s6_16657, s6_11807, s6_10067, s6_6928, s6_25858, s6_35189, s6_33576, s6_10826) and calcium transporters (s6_34277, s6_23789, s6_2349). Furthermore, transcripts encoding calmodulin (s6_2019 and s6_7832), which functions as a second messenger that regulates a myriad of vital biological processes, were reduced by more than threefold. These results suggest a potential impact on calcium homeostasis and the signal transduction pathways that it controls. Furthermore, although the precise reasons for the decreased expression of genes encoding the various ion transporters in SSB01 are not clear, they may integrate with Na^+^/Ca^2+^/K^+^-dependent signaling pathways, and their decline might cause severe perturbation of various physiological processes in the cells leading to increased oxidative stress and greater susceptibility to oxidative stress; terrestrial plants with reduced Ca^2+^ and K^+^ levels exhibited increased susceptibility to photo-oxidative damage [72, 73]. Additionally, transcripts encoding aquaporins (s6_28816 and s6_28625) also showed a more than twofold reduction after 8 d of exposure to elevated temperature, further indicating a broad suppression of transport-related gene expression under heat stress, although only in SSB01.

### Reduction in SSB01 transcripts encoding photosynthetic functions corresponds to a decline in photosynthesis during heat stress

After 4 d of heat stress, SSB01 exhibited significant downregulation of 15 transcripts related to photosynthesis (Fig. 4D). Among these, the transcripts encoding cytochrome *b_6_f* complex subunit 4 (*petD*) and the Photosystem II (PSII) CP43 antenna protein (*psbC*) were the most highly depleted, with each showing a greater than 6-fold decline in accumulation after 8 d of heat stress (Fig. 4D). Similarly, transcripts encoding the PSII proteins D2 (PsbD) and D1 (PsbA) declined by greater than five-fold following the imposition of heat stress (Fig. 4D). It was also shown in plants that heat stress elicits the formation of aggregates containing D1, CP43 and D2 and dysfunction of the photosynthetic machinery, which requires energy for protein turnover and repair/reassembly [74–77]. These findings align with observations of increased expression of proteasome complex subunits in SSB01 under elevated temperatures (Fig. 4A).

Additionally, the experiment revealed a >4-fold decrease in transcripts for the Photosystem I P700 chlorophyll *a* apoproteins A1 and A2 (PsaA and PsaB) after 8 d of heat exposure (Fig. 4D). The PSI complex core proteins, chlorophyll *a* apoproteins, are crucial for the integration of chlorophyll molecules into PSI. A heat-induced reduction in both *psbA* and *psaB* transcripts in SSB01 (not significantly changed in SSA01) suggests compromised synthesis of core reaction center proteins of both PSII and PSI, a phenomenon previously noted in other heat-sensitive Symbiodiniaceae species [78, 79].

Because construction of the RNA-seq library uses a selection for polyA RNA, it is biased toward the capture of nuclear-encoded transcripts (most chloroplast transcripts would be lost). RT-qPCR using gene specific primers and a total RNA template provides a more accurate measure of dynamic changes in accumulation of chloroplast transcripts. Therefore, to quantify levels of transcripts from chloroplast genes encoding proteins involved in photosynthesis, we performed RT-qPCR on eight representative target transcripts (*psbA*, *psbB*, *psbC*, *psbD*, *petB*, *petD*, *psaA, and psaB*), all of which appeared to be downregulated based on our RNA-seq results. Total RNA from SSB01 derived from cells maintained at 27°C or exposed to 34°C was randomly primed and reverse transcribed to synthesize cDNAs; the levels of individual transcripts were quantified based on qPCR from this cDNA library. Under control conditions (27°C), the levels of these transcripts remained stable across 0, 2, and 4 d, except for *psaB* and *psbD* transcripts, which appeared to increase to some extent (Fig. 4E). Upon heat stress (34°C), however, the transcript abundances from the 8 target transcripts all declined (Fig. 4F). In SSA01, the levels of these transcripts also declined following the 34°C treatment, but not to the same extent (Fig. S4). Additionally, with the marked reduction of chloroplast transcripts encoding components of the photosynthetic apparatus in SSB01, transcripts from nuclear-encoded genes associated with pigment biosynthesis and redox regulation, including geranylgeranyl reductase (s6_15050, *GGR*), phytoene desaturase (s6_14400, *PDS*), and thiol-disulfide oxidoreductases (s6_17805, DCC family) also exhibited reduced expression upon exposure to heat stress, albeit to a lesser extent than the chloroplast-encoded photosystem genes (Fig. 4D). GGR and PDS are key enzymes for chlorophyll and carotenoid biosynthesis, whereas DCC thiol-disulfide oxidoreductases facilitate protein folding and maintenance of cellular redox balance [80–82]. Downregulation of these nuclear-encoded transcripts further highlights the systemic disruption of photosynthesis and redox-protective processes in SSB01 during heat stress.

The photo-physiological responses in SSB01 and SSA01 at the elevated temperature were consistent with transcriptional alterations identified in these algae. SSB01 exhibited an initial sharp decrease in photosynthetic performance, as indicated by its *F*_v_/*F*_m_, which was followed by a more gradual steady decline over subsequent days, with highly reduced levels of chlorophyll *a* by d 4 (Fig. 4G and H). These observations suggest a pronounced susceptibility of SSB01 to thermal stress, impacting both photosynthetic efficiency and chlorophyll content. In contrast, SSA01 displayed a relatively small decline in both *F*_v_/*F*_m_ and chlorophyll *a* levels over the 4 d duration of the experiment (Fig. 4G and H). The stability of the photosynthetic apparatus aligns with the long-term persistence of DETs observed in SSA01 under elevated temperatures, underscoring its more robust tolerance of heat stress conditions compared to SSB01.

### Early differential expression in response to heat stress may confer thermal tolerance to SSA01

SSA01 responds rapidly to heat stress, displaying differential expression of hundreds of genes within the first 12 h of heat stress (Fig. S5). Upregulated genes encode proteins related to DNA repair and responses to stress conditions, whereas downregulated genes encode proteins related to post-transcriptional and post-translational modifications and complex sugar metabolism. These biological functions were not enriched in the transcriptomic response of SSB01 (Fig. 3E and F), raising the possibility that increased DNA repair, reduced RNA modifications and catabolism of complex sugars contribute to the heat tolerance of SSA01.

Oxidative stress related genes upregulated within 12 h of shifting SSA01 to 34°C encode a telomere elongation helicase 1 (RTEL1, SSA01_DN33433_c0_g1_i1) and an ascorbate peroxidase 1-like protein (APX1-like; APL, SSA01_DN66188_c0_g1_i1). RTEL1 is involved in DNA telomere-length regulation, DNA repair, and maintenance of genomic stability [83]. The *RTEL1* transcript is upregulated by ~1.5-fold after 3 h of exposure of the cells to the elevated temperature; this increased accumulation is maintained throughout the remainder of the heat treatment. Repairing damaged DNA, reinforcing stability of the genome immediately following exposure of cells to elevated temperatures, and maintaining these processes over an extended time could be important for sustaining the alga’s heat tolerance. The *APL* transcript is markedly upregulated during the early phase of heat exposure, and its level continues to increase over the duration of the experiment. Ascorbate peroxidases scavenge hydrogen peroxide in plants and play a critical role in the acclimation of *Arabidopsis thaliana* to abiotic stressors [84]. Intracellular peroxides and other ROS have been shown to accumulate in Symbiodiniaceae when experiencing heat stress [85]. These reactive metabolites would potentially be “quenched” by elevated levels of APL (and other antioxidant proteins).

Various transcripts related to complex sugar metabolism and post-transcriptional and post-translational modifications were downregulated within 12 h of exposing SSA01 to elevated temperatures. For example, beta galactosidase (*β-gal*, SSA01_DN32373_c0_g1_i1), glycosyl hydrolase (*GH*, SSA01_DN12486_c0_g3_i1), polygalacturonase (*PG*, SSA01_DN2449_c0_g1_i1), a putative glucan 1,3-beta-glucosidase A (*BGLA*, SSA01_DN3965_c0_g2_i1), a putative sulfate transporter *YbaR* (SSA01_DN4619_c0_g1_i1), lactoylglutathione lyase (*GloA*, SSA01_DN14325_c0_g1_i1), and 12-oxophytodienoate reductase 2 (*OPR*, SSA01_DN40576_c0_g1_i1), all transcripts involved in the metabolism of complex sugar, were downregulated during the early stages of heat stress. Accumulation of complex sugars in plants was observed after exposure to heat stress; these sugars helped maintain cell turgor, stabilize cell membranes and prevent protein degradation. They can also act as ROS scavengers to quench cellular damage that occurs when the cells are stressed [86]. Reduced complex sugar metabolism could be advantageous for Symbiodiniaceae experiencing elevated temperatures as these sugars could serve as energy reserves or in the modification of cell wall composition, aiding in the maintenance of homeostasis under stressful conditions. Because this study was conducted with axenic Symbiodiniaceae cultures, the metabolic responses observed here reflect algal cell–intrinsic regulation. However, bacterial associates are known to influence Symbiodiniaceae nutrient exchange and redox metabolism [87, 88], and could further modulate thermal and metabolic responses *in hospite*.

Transcripts encoding proteins involved in the transfer of acyl, methyl, and glycosyl groups to mRNA, tRNA, and proteins were significantly reduced during the early stages of heat stress in SSA01 (Dataset S2). Global reduction of post-transcriptional and post-translational modifications in heat tolerant SSA01 suggests a genome-wide suppression of gene/protein activities and reduced translation of proteins. Reduced biosynthetic/metabolic activities of the cells might diminish accumulation of misfolded or damaged proteins caused by the elevated temperature, enabling a more robust heat stress response and the allocation of resources toward repair of damaged DNA and proteins. This pattern was also reported for *Mesorhizobium loti*, a relatively heat tolerant species of symbiotic bacteria associated with plant roots. Exposure of *M. loti* to 30 min of elevated temperature (48°C) resulted in down regulation of 72% of the differentially expressed genes [89]. In contrast, transcripts associated with tRNA metabolic processes, protein methylation, RNA processing, and nucleic acid metabolism were upregulated in SSB01 after 8 d of heat stress. These increases in the metabolism of nucleic acids correlate with the dramatic transcriptomic responses of SSB01, suggesting that the global upregulation of various stress-related genes may be detrimental to the maintenance of heat tolerance or reflect an extreme response of the cells to cope with the stress conditions. In summary, SSA01 is likely successful at tolerating thermal stress, at least in part, as a consequence of its enhanced ability to quench oxidative damage and repair damaged biomolecules, which would limit their accumulation and reduce intracellular protein aggregation.

### Differential expression of orthologous genes in SSA01 and SSB01 provides insights into thermal tolerance

**Figure 5:**
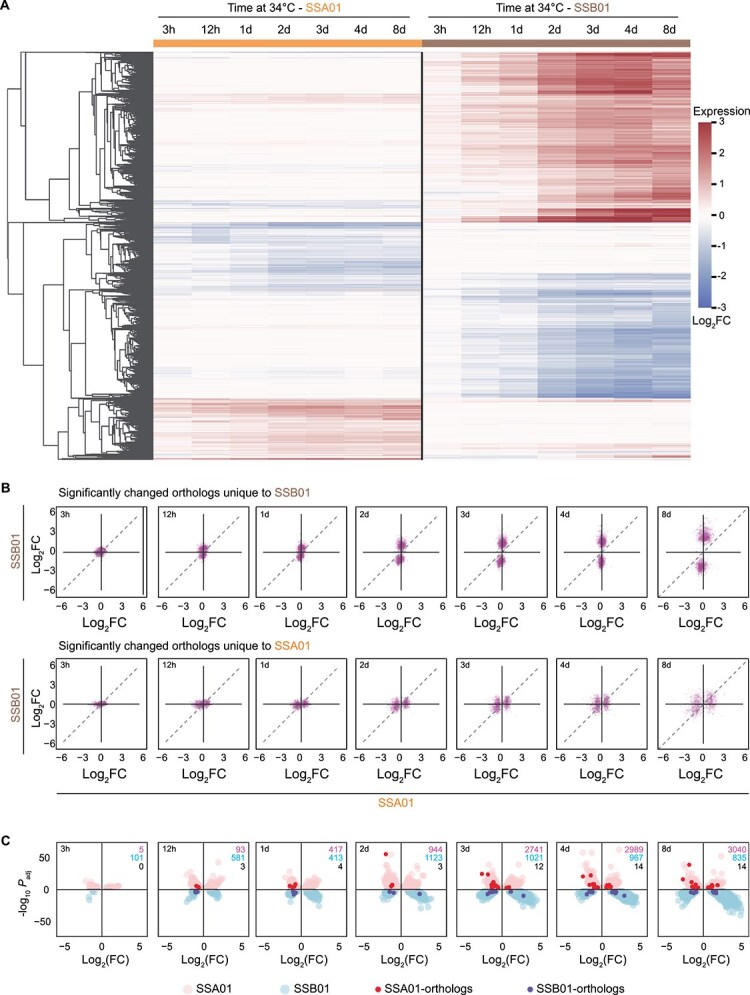
Orthologs with significant expression differences between SSA01 and SSB01. (A) Heatmap illustrating gene expression profiles of orthologs between SSA01 and SSB01 across various time points when exposed to an elevated temperature of 34°C. Each row of the *y*-axis represents an individual orthologous transcript, whereas the *x*-axis shows time points at which the samples were collected and analyzed. (B) Comparative scatter plot illustrating gene expression profiles of orthologs between SSB01 and SSA01 across various time points following exposure to 34°C. Top panels represent significantly changed levels of orthologous transcripts unique to SSB01, and the bottom panels represent significantly changed levels orthologous transcripts unique to SSA01. Each panel corresponds to a specific time point, with the *x*-axis displaying expression levels in SSA01 and the *y*-axis in SSB01. Each dot represents an ortholog that has exhibited significant expression changes in at least one of the species. This analysis facilitates direct comparison of gene expression between the two species across various time points. The grey dashed line denotes the line of equality, where the expression levels of orthologs are identical in both species. (C) Dual volcano plots depicting temporal changes in transcript abundances for SSA01 (top, pink) and SSB01 (bottom, blue) under elevated temperatures of 34°C. “SSA01-orthologs” and “SSB01- orthologs” denote orthologous genes shared between the two species that were significantly differentially expressed (adjusted *P* < 0.001). These orthologs are subsets of the total DETs for each species and are highlighted to illustrate that only a small proportion of shared orthologs display similar expression changes between the two lineages. Numbers in the upper-right corner of each plot indicate DETs unique to SSA01 (pink), unique to SSB01 (blue), and shared orthologs significantly changed in both species (black).

A rapid transcriptomic response during the initial hours of exposure of SSA01 to 34°C is followed by stabilization of gene expression over longer exposure times. In contrast, SSB01 exhibited a muted initial transcriptional response, with significant transcriptomic changes occurring after ~3 d at elevated temperatures. To investigate transcriptomic alterations among shared SSA01 and SSB01 genes during heat stress, we identified orthologs (Dataset S4) and analyzed their expression across all treatment times (Dataset S5). This analysis revealed distinct expression profiles associated with heat stress, with specific orthologs often differentially expressed between SSB01 or SSA01 (Fig. 5A). Following 8 d of exposure to 34°C elicited expression patterns of SSB01 that were generally more extreme than those of the orthologous genes in SSA01 (Fig. 5B). This difference in gene expression may reflect a difference in the ability of these Symbiodiniaceae to cope with the elevated temperature. The most significantly altered transcript levels were unique to each species, with few orthologs showing significant changes in both strains across all time points. By the eighth day of heat exposure, only 14 orthologs displayed a similar expression pattern (Fig. 5C), which is reflected in the dual volcano plots as a more pronounced downregulation of SSA01 expression compared to more subtle changes in SSB01 expression.

Comparative transcriptomic analyses of coral holobionts likewise show that Symbiodiniaceae from distinct genera exhibit largely species-specific transcriptomic responses to heat stress [49], paralleling our observation of limited overlap in differentially expressed orthologs between *S. linucheae* (SSA01) and *Breviolum minutum* (SSB01)*.* Early changes in the SSA01 expression pattern—particularly for those genes that rapidly respond to thermal stress and encode proteins involved in oxidoreductase activity, DNA/RNA binding, and homeostatic processes—may be key to its enhanced thermotolerance. Previous transcriptomic work aligns with our observation that early transcriptional shifts under heat stress often target genome organization and oxidative stress pathways. In *Durusdinium trenchii*, short-term exposure to elevated temperature (34°C for 6 h) induced differential expression of transcripts involved in chromatin organization and oxidative stress responses [90], supporting our model that rapid but targeted modulation of genome regulatory processes may contribute to thermal resilience. Nonetheless, given the phylogenetic and ecological diversity of the family [11], the specific molecular mechanisms that support thermotolerance likely differ among lineages, and broader comparative studies will be required to determine the extent to which these patterns are conserved.

We also examined orthologous transcripts that were differentially expressed in SSA01 after short exposures to elevated temperature (≤24 h), during which SSB01 exhibited minimal transcriptional changes (Fig. S5). Of these genes, there are many clusters (1, 4, 5, 7, 10, and 11) that exhibit opposite expression patterns in the two species. For example, cluster 1 is enriched in genes encoding methyltransferases and enzymes involved in tRNA modification. These genes are increasingly upregulated in SSB01 and downregulated in SSA01 over the entire 8 d exposure to the elevated temperature. These changes could cause alterations in transcriptional, translational and post-translational modifications that lead to a dramatic increase in the number of SSB01 DETs in cells that experienced 3 d or more of heat stress. Upregulated DETs in SSA01 are associated with DNA repair (Dataset S5).

During long-term heat exposure (3 d, 4 d, and 8 d), SSB01 exhibited significant differential expression (*P* <  0.001) of numerous transcripts, reflecting a sustained stress response to elevated temperature, in contrast to the relatively stable expression of orthologous genes in SSA01 over the same period (Dataset S5). These results are congruent with SSB01 having a delayed transcriptomic response to heat stress, with many genes orthologous to those of SSA01 displaying either upregulated or downregulated over the long term (Fig. 5). Among the most upregulated genes in SSB01 was a 2OG-Fe oxygenase (s6_44147), which shows homology to EGL nine-like protein 1 (EGLN1)—a cellular oxygen sensor that uses Fe(II) and 2-oxoglutarate to catalyze oxidative reactions linked to oxygen availability. Although both this enzyme and the JMJ21-like histone demethylase belong to the Fe(II)/2-oxoglutarate (2OG)-dependent dioxygenase superfamily, they act on distinct substrates and perform different biological functions. Upregulation of this EGLN1-like oxygenase under prolonged heat exposure may indicate activation of an oxygen-sensing pathway in response to sustained low-oxygen conditions caused by the decline in photosynthetic activity at the elevated temperature (Fig. 4H). This change in expression could be an adaptive mechanism that helps SSB01 cope with hypoxic stress arising from thermal damage to the photosynthetic apparatus. A comparable response occurs in *Fugacium kawagutii*, which under heat stress downregulates PSI reaction center subunit IV and the iron permease (*FTR1*) but upregulates phytoglobin and inositol oxygenase [60], consistent with activation of oxygen-responsive pathways. This parallel raises the possibility that modulation of oxygen-related metabolism may be a broader Symbiodiniaceae strategy during thermal stress. The functional diversity of this enzyme family in Symbiodiniaceae is further illustrated by our recent identification of a chlorophyll *c* synthase (CHLCS) that contains both chlorophyll *a*/*b*-binding and 2OG-Fe(II) dioxygenase (2OGD) domains [91]. The 2OGD domain catalyzes chlorophyll *c* biosynthesis, underscoring how 2OG-Fe(II) oxygenases contribute not only to stress signaling and epigenetic regulation but also to core photosynthetic pigment production. Together, these findings highlight the metabolic and functional versatility of 2OG-Fe(II) oxygenases in Symbiodiniaceae and their potential roles in redox regulation during heat stress. The other highly upregulated transcripts in SSB01 include a vacuolar protein sorting-associated protein VTA1-like (s6_22979), and chloroplastic pentatricopeptide repeat-containing protein (s6_47105). The most downregulated genes are associated with peptidase activity, including DASH (Drosophila, Arabidopsis, Synechocystis, Human)-type cryptochromes (s6_52040), and signal peptide peptidase-like 3 (s6_7428) (Dataset S5). This suggests that SSB01 may have reduced protein degradation following heat stress, even though many of these proteins would likely suffer oxidative damage. Accumulation of damaged cellular components could be a major factor in heat sensitivity.

Beginning on d-3 of heat stress, genes exhibiting the highest differential expression in SSB01 were nearly all upregulated, with most by >2-fold. All of the most upregulated genes (>8-fold increase in expression) have not been well-characterized, however, GO terms that were enriched at the later time points (3 days and longer) in SSB01 included glycerol and glycerol-3-phosphate metabolic process [including glycerol kinase (s6_12670); upregulated >5-fold after 3 d at 34°C], and alditol and polyol metabolic processes. Similar alterations in carbon metabolism were reported in a study tracking the fate of carbon during coral bleaching [92]. Metabolomic analyses demonstrated an increase in glycerol abundance in dinoflagellates exposed to long-term heat stress (6 and 9 d), with a potential increase in metabolic pathways that generate glycerol. These metabolic adjustments may help compensate for reduction in the generation of photosynthesis-derived energy and fixed carbon, thereby prolonging survival of the endosymbionts during heat stress. Future mechanistic studies will be necessary to test this possibility and clarify the role of glycerol metabolism in thermal resilience.

## Conclusion

**Figure 6:**
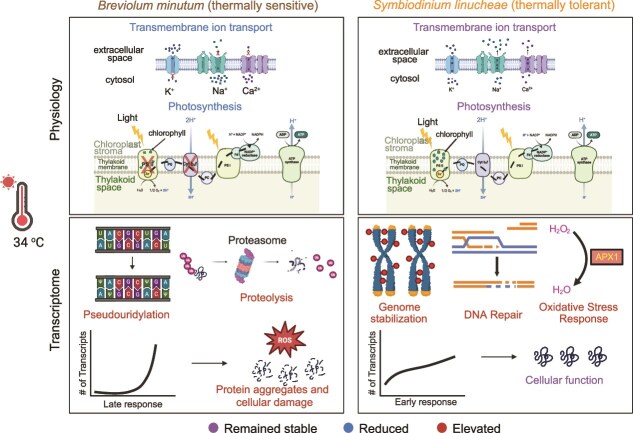
Model of molecular basis of Symbiodiniaceae thermal sensitivity and tolerance. Illustrations of differential responses of *Symbiodinium linucheae* SSA01 and *Breviolum minutum* SSB01 to elevated temperatures. At 34°C, the transcriptome of SSB01 exhibits stress-induced modifications that include decreased photosynthetic function and cellular transport and increased nucleic acid modifications and proteasome activity. This stress response is delayed (relative to SSA01) and leads to accumulation of cell damage and dysfunctional protein aggregates that elicit further long-term transcriptomic alterations. These include downregulation of photosynthesis and cellular transport, upregulation of proteolysis, and ultimately cell death. In contrast, SSA01 initiates an early transcriptional response involving genome stabilization, DNA repair, and oxidative-stress mitigation occurring through mechanisms such as APX1-mediated ROS scavenging. This rapid activation of protective pathways and controlled transcriptional modulation supports cellular stability and sustained function during prolonged heat exposure. Together, these data highlight a thermotolerance strategy in SSA01 that contrasts with the stress-driven, damage-associated responses observed in SSB01. Figure created in part using BioRender.com.

We characterized the physiological and transcriptional responses of two Symbiodiniaceae species, *Symbiodinium linucheae* SSA01 and *Breviolum minutum* SSB01, to thermal stress. We demonstrate that SSA01 is more thermally tolerant than SSB01, both *in hospite* and in culture. SSB01 in culture exhibited a heat stressed transcriptome that correlated with a reduction in photosynthetic activity and ion/metabolite transport, and an increase in nucleic acid modifications and proteasome activity. In contrast, SSA01 mounts a rapid transcriptional response to elevated temperature and was able to maintain cellular function throughout the 8 d of exposure to 34°C. Because SSB01 did not mount a short-term response to heat exposure, cellular damage and dysfunctional protein aggregates likely formed at a high rate during the first few days of exposure to the elevated temperature, leading to long-term transcriptomic changes that reflect marked alterations in metabolism, including down-regulating photosynthesis, transport processes and the upregulating glycerol metabolism, DNA stabilization, and proteolysis. The rapid short-term response of SSA01 to heat exposure led to the down-regulation of transcripts associated with potentially damaging cellular processes and upregulating proteolysis (e.g. removal of damaged proteins) and other regulatory mechanisms that could potentially confer thermal tolerance to this species (Fig. 6). These results provide insights into molecular mechanisms responsible for thermal plasticity among the different Symbiodiniaceae endosymbionts, which is crucial for understanding how coral reefs will respond to global warming and rising seawater temperatures.

## Supplementary Material

Heat_Transcriptome_Supplemental_2025_11_30_wraf268(1)

Dataset-S1_wraf268

Dataset-S2_wraf268

Dataset-S3_wraf268

Dataset-S4_wraf268

Dataset-S5_wraf268

## Data Availability

All raw sequencing reads of *Breviolum minutum* strain SSB01 and *Symbiodinium linucheae* strain SSA01 under different temperature conditions are available in the Sequence Read Archive (http://www.ncbi.nlm.nih.gov/sra) with accession number PRJNA591730.
