# Supplementary material for: Distinct transcriptomic strategies underlie differential heat tolerance in Symbiodiniaceae symbionts
Source: ISME J. 2025 Dec 4;19(1):wraf268. doi: 10.1093/ismejo/wraf268 (PMC12743301; doi:10.1093/ismejo/wraf268)
Supplement: Heat_Transcriptome_Supplemental_2025_11_30_wraf268(1) [file heat_transcriptome_supplemental_2025_11_30_wraf268(1).docx]

*Supplementary material*

Distinct transcriptomic strategies underlie differential heat tolerance in Symbiodiniaceae symbionts

Tingting Xiang, Stephanie L. Peak, Eric C. Huitt, Arthur R. Grossman


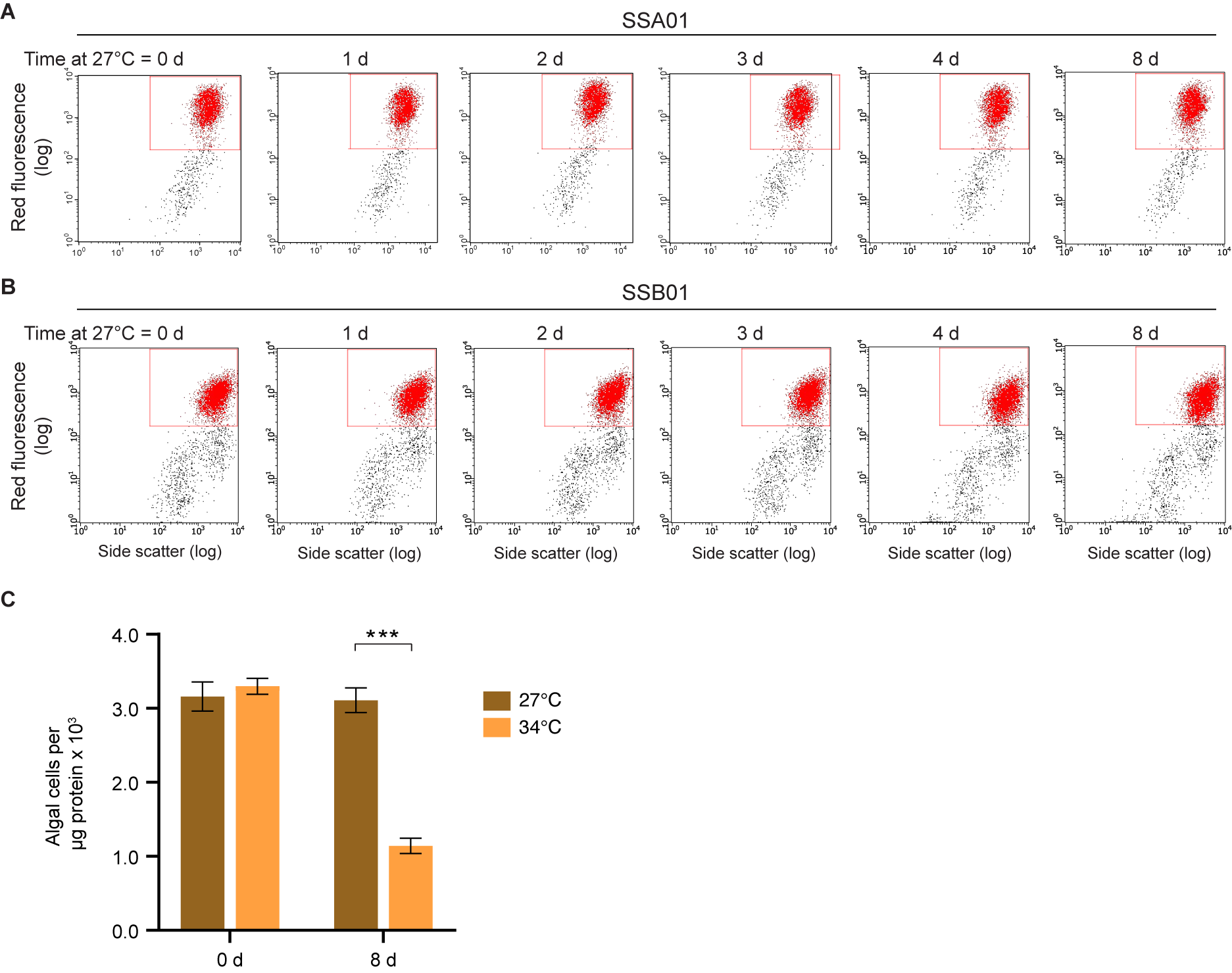


**Figure S1. SSA01 and SSB01 maintain stable chlorophyll fluorescence under control conditions.**

Flow cytometry profiles showing chlorophyll fluorescence and side-scatter for cultured *S. linucheae* SSA01 **(A)** and *B. minutum* SSB01 **(B)** maintained at ambient (control) temperature (27 °C) for 8 d in parallel with the heat-treated (34 °C) cultures (shown in **Fig. 1**). Each dot represents an individual cell. Red populations (within the fluorescence-side scatter window) represent healthy cells. **(C)** SSB01 Symbiont density in Aiptasia (CC7) at 27 °C and 34 °C at 0 d and 8 d post-heat exposure. Symbiont abundance was normalized to host protein content (algal cells per µg host protein). Error bars represent mean ± s.e.m. of 8 biological replicates. ****P* < 0.001.

**
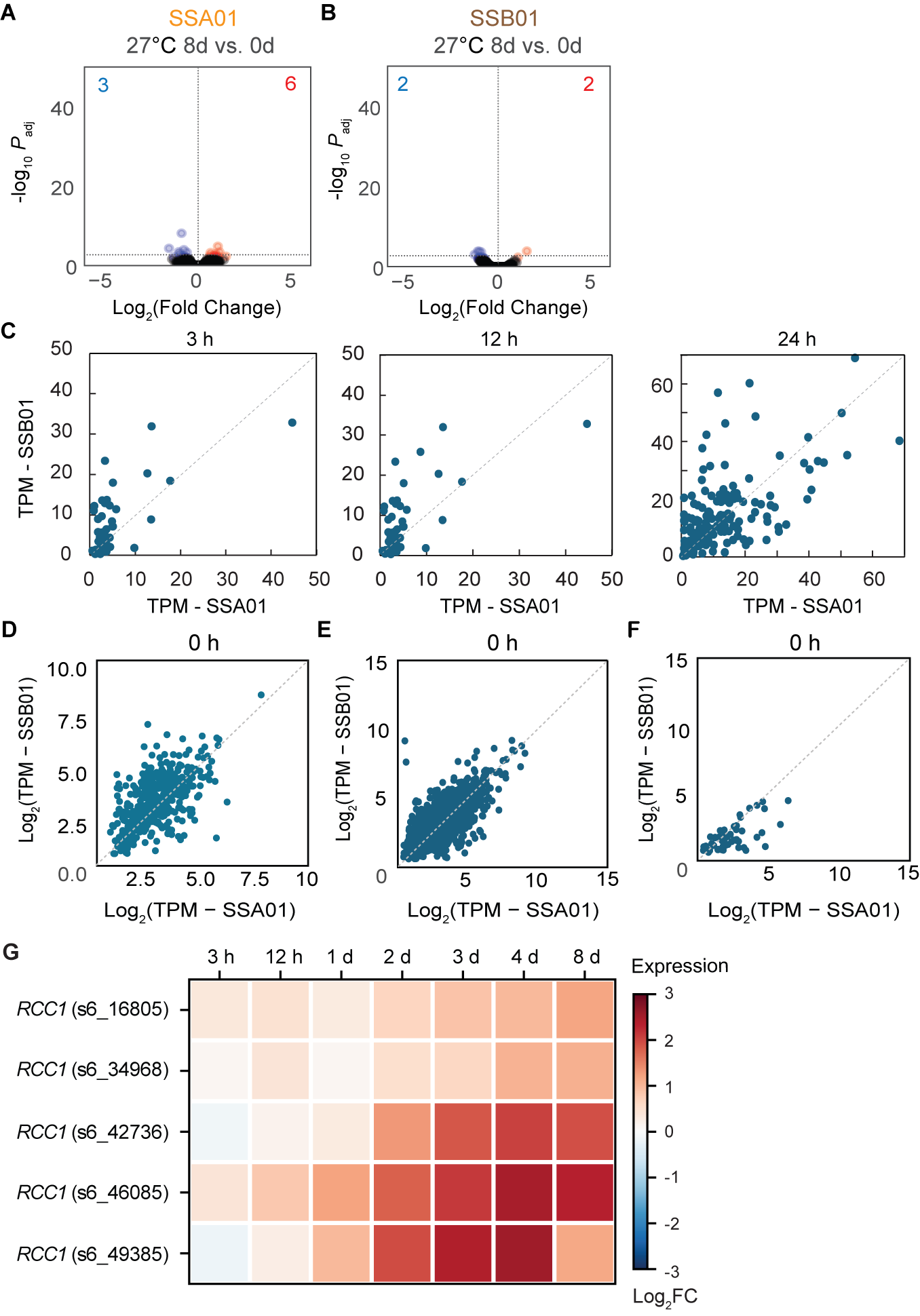
**

**Figure S2. Transcriptomic comparison of SSA01 and SSB01 under control conditions and early heat stress.**

(**A**, **B**) Volcano plots showing DETs in SSA01 (**A**) and SSB01 (**B**) after 8 d at 27 °C relative to time 0. Very few DETs were identified, indicating minimal transcriptomic changes under control conditions. Numbers in the upper corners denote upregulated (red) and downregulated (blue) transcripts. (**C**) Scatter plots comparing transcript abundance (TPM) between SSA01 and SSB01 at 3 h, 12 h, and 24 h after heat stress exposure (34 °C). Transcripts that were differentially expressed in SSA01 are shown. **(D)** Scatter plot comparing TPMs at 0 h for SSA01 heat-responsive transcripts between SSA01 and SSB01. **(E)** TPMs at 0 h for SSB01 transcripts identified as heat-elevated during short-term heat stress (3 h, 12 h, 24 h) and their SSA01 orthologs, compared between the two species. **(F)** TPMs at 0 h for high (Log_2_FC > 2) heat-elevated SSB01 transcripts and their SSA01 orthologs. Blue dots indicate unique transcripts; dashed diagonal lines represent equal expression between species. (**G**) Heatmap showing log₂ fold change (Log_2_FC) of transcripts encoding Regulator of Chromosome Condensation 1 (RCC1)–family proteins in SSB01 across time points at 34 °C relative to 0 h, revealing coordinated upregulation during prolonged heat exposure.


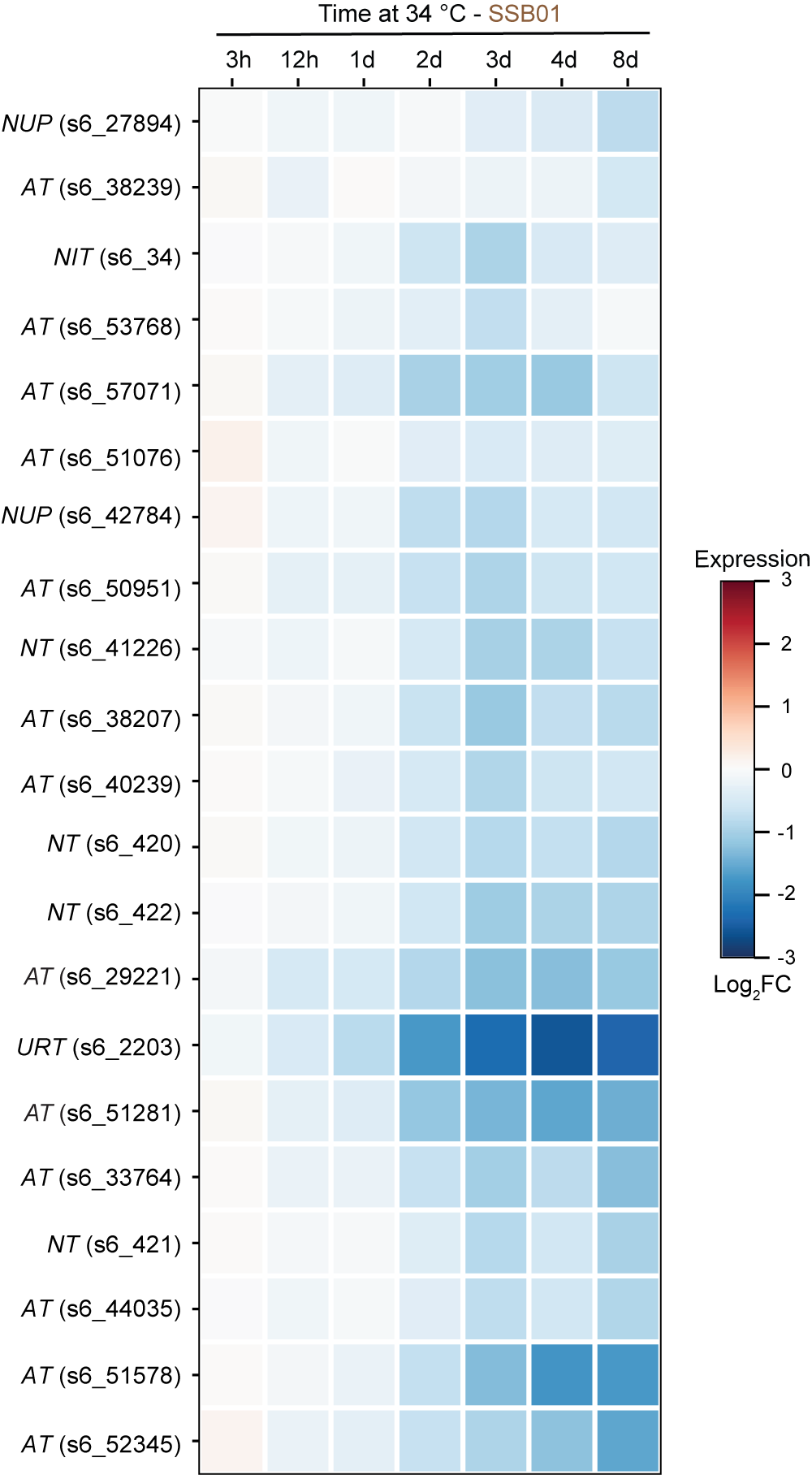


**Figure S3. Expression of nitrogen-acquisition transcripts in SSB01 under heat stress.**

Heatmap showing log₂ fold changes (Log_2_FC) of transcripts encoding nitrogen-acquisition genes in SSB01 exposed to 34 °C relative to 27 °C, including putative nitrate (NT), ammonium (AT), adenine/guanine (NUP), and urea (URT) transporters, and a putative nitrate reductase (NIT).


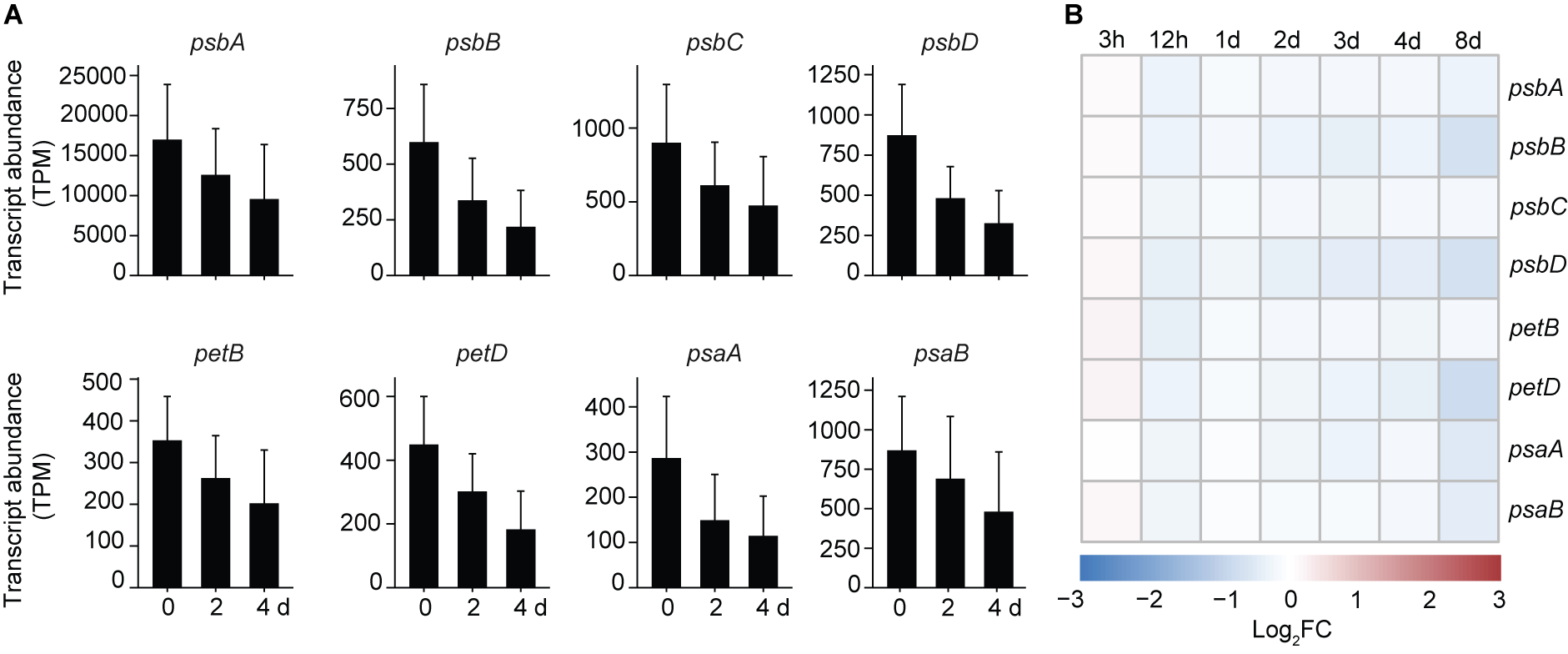


**Figure S4. Transcript levels and differential expression of chloroplast genes in SSA01 under heat stress.**

(**a**) Transcript levels (measured by transcripts per million, TPM) of select chloroplast genes at 0, 2, and 4 d of heat exposure (34 °C). Bars represent the mean TPM ± SE of at least 3 biological replicates. Although some decline in transcript levels was observed, these changes in TPM were statistically not significant across the days (*P* > 0.05). (**b**) Heatmap showing differential expression of chloroplast genes (measured by log_2_ fold change, Log_2_FC) after 3 h, 12 h, 1 d, 2 d, 3 d, 4 d, and 8 d at 34 °C.


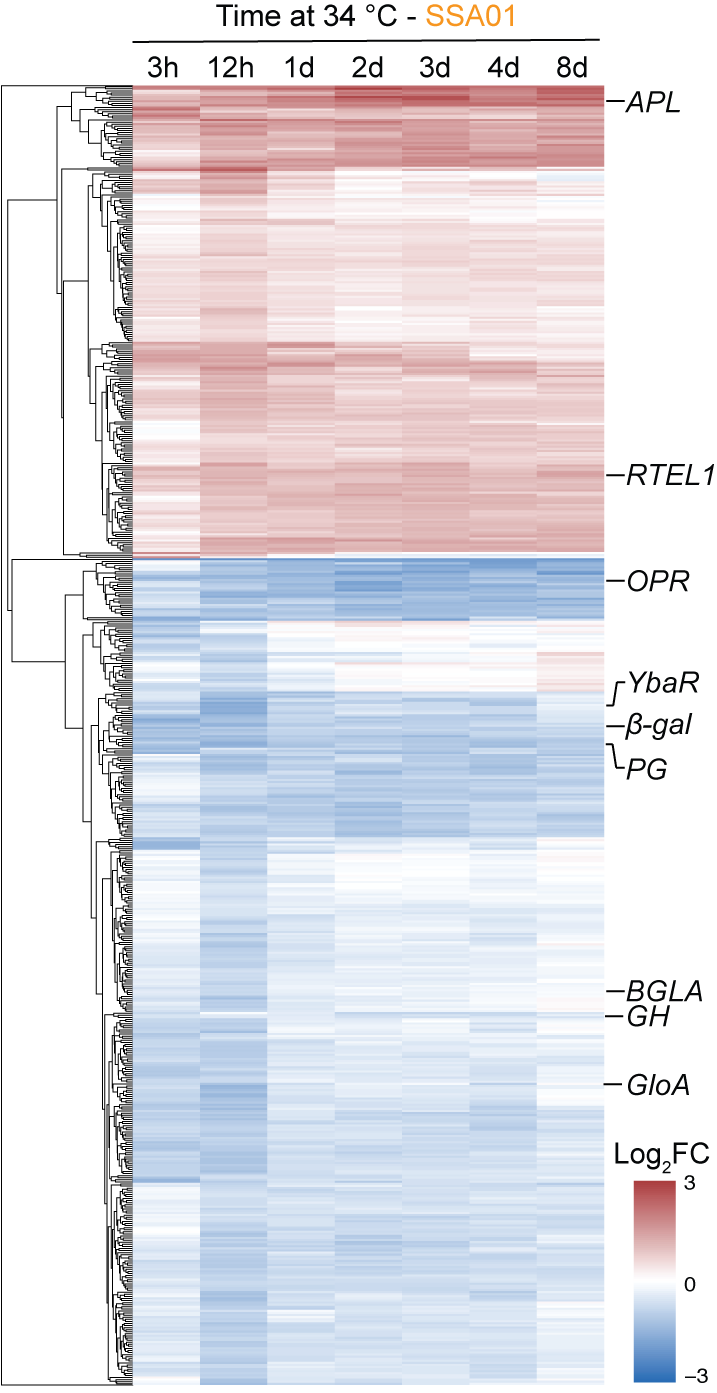


**Figure S5. Differentially expressed genes in SSA01 within 12 h of heat stress.**

A total of 584 transcripts were significantly differentially expressed (*P* < 0.001, fold change at 34 °C relative to 27 °C) in SSA01 during the initial 12 h of exposure to the elevated temperature. The heat map shows expression patterns of these transcripts across all time points from 3 h to 8 d. Each row represents an individual SSA01 transcript. Log₂ fold change values indicate relative expression at 34 °C compared to time 0 (27 °C just prior to the change of temperature). Labeled genes highlight functional categories including oxidative stress response (*APL*, *RTEL1*), protein quality control (*OPR*, *YbaR*), metabolic activity (*β-gal*, *PG*, *GloA*), and carbohydrate processing (*BGLA*, *GH*).

**Table S1. RNA-seq library metadata for SSA01 and SSB01 samples across all time points.**

| **Species** | **Temperature (°C)** | **Time (h)** | **Library Name** | **Library Barcode** |
| --- | --- | --- | --- | --- |
| SSB01 | 34 | 0 | 151020_MONK_0446_AC7LDNACXX_L1 | ATCACG |
| SSB01 | 34 | 0 | 151020_MONK_0446_AC7LDNACXX_L1 | GATCAG |
| SSB01 | 34 | 0 | 140319_TENNISON_0286_AC3L8RACXX_L3 | CGATGT |
| SSB01 | 34 | 3 | 151020_MONK_0446_AC7LDNACXX_L1 | TTAGGC |
| SSB01 | 34 | 3 | 151020_MONK_0446_AC7LDNACXX_L1 | ACTTGA |
| SSB01 | 34 | 3 | 140319_TENNISON_0286_AC3L8RACXX_L3 | TGACCA |
| SSB01 | 34 | 12 | 151020_MONK_0446_AC7LDNACXX_L1 | TAGCTT |
| SSB01 | 34 | 12 | 151020_MONK_0446_AC7LDNACXX_L1 | GGCTAC |
| SSB01 | 34 | 12 | 140402_TENNISON_0288_AC3KVVACXX_L6 | CGATGT |
| SSB01 | 34 | 24 | 151020_MONK_0446_AC7LDNACXX_L1 | GTGGCC |
| SSB01 | 34 | 24 | 151020_MONK_0446_AC7LDNACXX_L1 | GTTTCG |
| SSB01 | 34 | 24 | 140319_TENNISON_0286_AC3L8RACXX_L3 | ACAGTG |
| SSB01 | 34 | 48 | 151020_MONK_0446_AC7LDNACXX_L1 | CGTACG |
| SSB01 | 34 | 48 | 151020_MONK_0446_AC7LDNACXX_L1 | GAGTGG |
| SSB01 | 34 | 48 | 140321_PINKERTON_0294_BC3K58ACXX_L1 | GCCAAT |
| SSB01 | 34 | 72 | 151020_MONK_0446_AC7LDNACXX_L1 | ACTGAT |
| SSB01 | 34 | 72 | 151020_MONK_0446_AC7LDNACXX_L2 | ATTCCT |
| SSB01 | 34 | 72 | 140402_TENNISON_0288_AC3KVVACXX_L6 | GCCAAT |
| SSB01 | 34 | 96 | 151020_MONK_0446_AC7LDNACXX_L2 | CGATGT |
| SSB01 | 34 | 96 | 151020_MONK_0446_AC7LDNACXX_L2 | TGACCA |
| SSB01 | 34 | 96 | 140402_TENNISON_0288_AC3KVVACXX_L6 | AGTCAA |
| SSB01 | 34 | 192 | 151020_MONK_0446_AC7LDNACXX_L2 | ACAGTG |
| SSB01 | 34 | 192 | 151020_MONK_0446_AC7LDNACXX_L2 | GCCAAT |
| SSB01 | 27 | 192 | 151020_MONK_0446_AC7LDNACXX_L2 | CGTACG |
| SSB01 | 27 | 192 | 151020_MONK_0446_AC7LDNACXX_L2 | GAGTGG |
| SSB01 | 27 | 192 | 151020_MONK_0446_AC7LDNACXX_L2 | ACTGAT |
| SSA01 | 34 | 0 | 151020_MONK_0446_AC7LDNACXX_L3 | CAGATC |
| SSA01 | 34 | 0 | 151020_MONK_0446_AC7LDNACXX_L3 | CTTGTA |
| SSA01 | 34 | 0 | 140319_TENNISON_0286_AC3L8RACXX_L3 | CAGATC |
| SSA01 | 34 | 0 | 140402_TENNISON_0288_AC3KVVACXX_L7 | AGTTCC |
| SSA01 | 34 | 3 | 151020_MONK_0446_AC7LDNACXX_L3 | ATGTCA |
| SSA01 | 34 | 3 | 151020_MONK_0446_AC7LDNACXX_L3 | CCGTCC |
| SSA01 | 34 | 3 | 140402_TENNISON_0288_AC3KVVACXX_L6 | GTCCGC |
| SSA01 | 34 | 12 | 151020_MONK_0446_AC7LDNACXX_L3 | GTCCGC |
| SSA01 | 34 | 12 | 151020_MONK_0446_AC7LDNACXX_L3 | GTGAAA |
| SSA01 | 34 | 12 | 140321_PINKERTON_0294_BC3K58ACXX_L1 | CTTGTA |
| SSA01 | 34 | 24 | 151020_MONK_0446_AC7LDNACXX_L3 | ATCACG |
| SSA01 | 34 | 24 | 151023_BRISCOE_0264_BC7LUPACXX_L1 | GATCAG |
| SSA01 | 34 | 24 | 140321_PINKERTON_0294_BC3K58ACXX_L1 | AGTCAA |
| SSA01 | 34 | 48 | 151023_BRISCOE_0264_BC7LUPACXX_L1 | TTAGGC |
| SSA01 | 34 | 48 | 151023_BRISCOE_0264_BC7LUPACXX_L1 | ACTTGA |
| SSA01 | 34 | 48 | 140402_TENNISON_0288_AC3KVVACXX_L7 | TGACCA |
| SSA01 | 34 | 72 | 151023_BRISCOE_0264_BC7LUPACXX_L1 | TAGCTT |
| SSA01 | 34 | 72 | 151023_BRISCOE_0264_BC7LUPACXX_L1 | GGCTAC |
| SSA01 | 34 | 72 | 140321_PINKERTON_0294_BC3K58ACXX_L1 | AGTTCC |
| SSA01 | 34 | 96 | 151023_BRISCOE_0264_BC7LUPACXX_L1 | GTGGCC |
| SSA01 | 34 | 96 | 151023_BRISCOE_0264_BC7LUPACXX_L1 | GTTTCG |
| SSA01 | 34 | 96 | 140402_TENNISON_0288_AC3KVVACXX_L7 | ACAGTG |
| SSA01 | 34 | 192 | 151023_BRISCOE_0264_BC7LUPACXX_L1 | ATTCCT |
| SSA01 | 34 | 192 | 151023_BRISCOE_0264_BC7LUPACXX_L1 | CGATGT |
| SSA01 | 34 | 192 | 140402_TENNISON_0288_AC3KVVACXX_L7 | CAGATC |
| SSA01 | 27 | 192 | 151020_MONK_0446_AC7LDNACXX_L3 | AGTCAA |
| SSA01 | 27 | 192 | 151020_MONK_0446_AC7LDNACXX_L3 | AGTTCC |

**FIGURE LEGENDS**

**Supplementary Figure 1. SSA01 and SSB01 maintain stable chlorophyll fluorescence under control conditions.**

Flow cytometry profiles showing chlorophyll fluorescence and side-scatter for cultured *S. linucheae* SSA01 **(a)** and *B. minutum* SSB01 **(b)** maintained at ambient (control) temperature (27°C) for 8 d in parallel with the heat-treated (34 °C) cultures (shown in **Fig. 1**). Each dot represents an individual cell. Red populations (within the fluorescence-side scatter window) represent healthy cells. **(c)** SSB01 Symbiont density in Aiptasia at 27 °C and 34 °C at 0 d and 8 d post-heat exposure. Symbiont abundance was normalized to host protein content (algal cells per µg host protein). Error bars represent mean ± s.e.m. of 8 biological replicates. ****P* < 0.001.

**Supplementary Figure 2**. **Transcriptomic comparison of SSA01 and SSB01 under control conditions and early heat stress.**

(**a**, **b**) Volcano plots showing DETs in SSA01 (**a**) and SSB01 (**b**) after 8 d at 27 °C relative to time 0. Very few DETs were identified, indicating minimal transcriptomic changes under control conditions. Numbers in the upper corners denote upregulated (red) and downregulated (blue) transcripts. (**c**) Scatter plots comparing transcript abundance (TPM) between SSA01 and SSB01 at 3 h, 12 h, and 24 h after heat stress exposure (34 °C). Transcripts that were differentially expressed in SSA01 are shown. **(d)** Scatter plot comparing TPMs at 0 h for SSA01 heat-responsive transcripts between SSA01 and SSB01. **(e)** TPMs at 0 h for SSB01 transcripts identified as heat-elevated during short-term heat stress (3 h, 12 h, 24 h) and their SSA01 orthologs, compared between the two species. **(f)** TPMs at 0 h for high (Log_2_FC > 2) heat-elevated SSB01 transcripts and their SSA01 orthologs. Blue dots indicate unique transcripts; dashed diagonal lines represent equal expression between species. (**g**) Heatmap showing log₂ fold change (Log_2_FC) of transcripts encoding Regulator of Chromosome Condensation 1 (RCC1)–family proteins in SSB01 across time points at 34 °C relative to 0 h, revealing coordinated upregulation during prolonged heat exposure.

**Supplementary Figure 3. Expression of nitrogen acquisition transcripts in SSB01 under heat stress.**

Heatmap showing log₂ fold changes (Log_2_FC) of transcripts encoding nitrogen-acquisition genes in SSB01 exposed to 34 °C relative to 27 °C, including putative nitrate (NT), ammonium (AT), adenine/guanine (NUP), and urea (URT) transporters, and a putative nitrate reductase (NIT).

**Supplementary Figure 4. Transcript levels and differential expression of chloroplast genes in SSA01 under heat stress.**

(**a**) Transcript levels (measured by transcripts per million, TPM) of select chloroplast genes at 0, 2, and 4 d of heat exposure (34 °C). Bars represent the mean TPM ± SE of at least 3 biological replicates. Although some decline in transcript levels was observed, these changes in TPM were statistically not significant across the days (*p* > 0.05). (**b**) Heatmap showing differential expression of chloroplast genes (measured by log_2_ fold change, Log_2_FC) after 3 h, 12 h, 1 d, 2 d, 3 d, 4 d, and 8 d at 34 °C.

**Supplementary Figure 5. Differentially expressed genes in SSA01 within 12 h of heat stress.**

A total of 584 transcripts were significantly differentially expressed (*p* < 0.001, fold change at 34 °C relative to 27°C) in SSA01 during the initial 12 h of exposure to the elevated temperature. The heat map shows expression patterns of these transcripts across all time points from 3 h to 8 d. Each row represents an individual SSA01 transcript. Log₂ fold change values indicate relative expression at 34 °C compared to time 0 (27 °C just prior to the change of temperature). Labeled genes highlight functional categories including oxidative stress response (*APL*, *RTEL1*), protein quality control (*OPR*, *YbaR*), metabolic activity (*β-gal*, *PG*, *GloA*), and carbohydrate processing (*BGLA*, *GH*).

**Table S1. RNA-seq library metadata for SSA01 and SSB01 samples across all time points.**

**Dataset S1. Differentially expressed transcripts in SSB01 across all time points under heat stress.**

**Dataset S2. Differentially expressed transcripts in SSA01 across all time points under heat stress.**

**Dataset S3. Transcript abundance (TPM) at 0 h prior to heat exposure for transcripts identified as heat-elevated in SSA01 or SSB01.**

**Dataset S4. Orthologous transcript pairs identified between SSA01 and SSB01.**

**Dataset S5. Differentially expressed orthologs between SSA01 and SSB01, along with their expression levels across all time points under heat stress.**
